# Prevalence of Metabolic Syndrome in Obese Children and Adolescents using Three Different Criteria and Evaluation of Risk Factors

**DOI:** 10.4274/jcrpe.v3i2.15

**Published:** 2011-06-08

**Authors:** Özlem Sangun, Bumin Dündar, Muhammet Köşker, Özgür Pirgon, Nihal Dündar

**Affiliations:** 1 Department of Pediatrics, Division of Pediatric Endocrinology, Faculty of Medicine, Suleyman Demirel University, Isparta, Turkey; 2 Department of Pediatrics, Division of Pediatric Endocrinology, Research and Training Hospital, Konya, Turkey; 3 Department of Pediatrics, Faculty of Medicine, Suleyman Demirel University, Isparta, Turkey

**Keywords:** Childhood metabolic syndrome, prevalence, risk factors, breastfed, puberty

## Abstract

Objective: To compare the prevalence of the metabolic syndrome (MS) in Turkish obese children and adolescents by using three different definitions and to assess the risk factors through a retrospective evaluation of anthropometric and laboratory parameters.

Methods: Sixty hundred and fourteen obese patients (307 male, 307 female; mean age: 11.3±2.5 years) were included in the study. Medical history, physical examination, anthropometric measurements, results of biochemical and hormonal assays were obtained from the hospital records. MS was diagnosed according to the modified World Health Organization (WHO), Cook and the International Diabetes Federation (IDF) consensus criteria.

Results: The prevalence of MS was found to be 39%, 34% and 33% according to the modified WHO, Cook and the IDF consensus criteria, respectively. MS prevalence in patients aged 12-18 years was significantly higher than that in patients between 7 and 11 years of age (p<0.05). Pubertal patients had a significantly higher MS prevalence than the non-pubertal cases (p<0.05). MS prevalence was also significantly higher in children who had a family history of heart disease, diabetes, obesity and hypertension as well as in those who had not been breast-fed (p<0.05).

**Conclusion:** The use of the modified WHO criteria was found to result in a slightly higher prevalence rate for MS as compared to the other criteria. The prevalence of MS in our study population was higher than that reported in most previous studies in Turkey. A positive family history, puberty and not being breastfed in infancy were shown to be significant risk factors for MS in childhood.

The prevalence of MS was found to be 39%, 34% and 33% according to the modified WHO, Cook and the IDF consensus criteria, respectively. MS prevalence in patients aged 12-18 years was significantly higher than that in patients between 7 and 11 years of age (p<0.05). Pubertal patients had a significantly higher MS prevalence than the non-pubertal cases (p<0.05). MS prevalence was also significantly higher in children who had a family history of heart disease, diabetes, obesity and hypertension as well as in those who had not been breast-fed (p<0.05).

**Conclusion:** The use of the modified WHO criteria was found to result in a slightly higher prevalence rate for MS as compared to the other criteria. The prevalence of MS in our study population was higher than that reported in most previous studies in Turkey. A positive family history, puberty and not being breastfed in infancy were shown to be significant risk factors for MS in childhood.

**Conflict of interest:**None declared.

## INTRODUCTION

Obesity in children and adolescence is on the increase due to changes in lifestyle and nutrition behaviors (1). Children who used to play outdoors in the past years are today more likely to spend their time in front of television and computer screens. It is known that 1/3 of obese children and 80% of obese adolescents remain obese when they reach adulthood (2). The World Health Organization (WHO) defines obesity as ‘‘abnormal or excessive fat accumulation that presents a risk to health’’ (3). Obese children are also more prone to have metabolic syndrome (MS), type 2 diabetes, hypertension, hyperlipidemia, cardiovascular disease and some types of cancer (colon, breast, gallbladder, endometrium) (4).

A set of risk factors associated with obesity and insulin resistance lead to chronic changes in various tissues and organs. There are racial differences in the prevalence of obesity and in the prevalence of obesity-related health complications. Although it is known that accurate management of  MS is fundamental in controlling  the current global epidemics of cardiovascular disease and diabetes mellitus, the predisposing risk factors or epidemiologic characteristics of MS are not yet clearly defined in children and adolescents (5).

The prevalence of MS in different countries has been reported to be 3-4% (6). Reports from different provinces in Turkey indicate that the prevalence of MS ranges around 3%, while it may be as high as 20-38% in obese children (7,8,9,10). In a recent study, the MS prevalence based on the International Diabetes Federation (IDF) guidelines was reported to be 2.3% in the total population. The prevalence was found to be similar by both IDF and National Cholesterol Education Program (NCEP) definitions, but it was higher when assessed according to the WHO definition (11). 

Although there is an increasing number of reports regarding childhood MS, there is still no universal agreement on which level, and even which criteria, should be used for the diagnosis of MS. The most commonly applied criteria recommended for use in comparative studies are the modified WHO, Cook and the IDF consensus criteria (12,13). The aim of this study was to determine the prevalence of and risk factors for MS in obese children and to compare the three definition criteria for the diagnosis of MS in children and adolescents.

## MATERIALS AND METHODS

Sixty hundred and thirty-five obese children and adolescents aged between 7 and 18 years were recruited from obese children who presented to the Pediatric Endocrinology Unit. The children were required to meet the following inclusion criteria: (1) age, 7-18 years; (2) a BMI greater than the 95th percentile for age and gender based on the standards of the Centers for Disease Control and Prevention (CDC); (3) absence of a prior major illness, including type 1 or 2 diabetes. Being on medications or having a condition known to influence body composition, insulin action or insulin secretion (e.g. glucocorticoid therapy, hypothyroidism and Cushing’s disease) was a reason for exclusion. Twenty-one cases were excluded due to inadequacy of the information in their files. Thus, 614 obese children (307 girls and 307 boys) were included in the study. 

The mean age of the group was 11.3±2.5 years (range: 7-18 years) and the mean body mass index standard deviation score (BMI-SDS) was 2.57±0.59.  Data on age, gender, birth weight, and feeding in the first 6 months (regarding exclusive breast-feeding) were obtained from the medical records. Cardiovascular disease, type 2 diabetes, obesity and hypertension in the family (first and second degree relatives) were also recorded from the specifically asked questions in the patient reports.

Height and weight were measured by the same pediatrician. Height was measured to the nearest 0.5 cm on a standard height board, and weight was determined to the nearest 0.1 kg on a standard physician’s beam scale with the subject dressed only in light underwear and no shoes. BMI was calculated as weight in kilograms divided by height in meters squared. All measuring devices were weekly calibrated. Weight SDS, height SDS, BMI, and BMI-SDS were calculated for all patients. The degree of obesity was quantified using the Cole’s least mean square method, which normalizes the BMI skewed distribution and expresses BMI as an SDS (BMI-SDS). This measure gives age- and sex-specific estimates of the distribution median, the coefficient of variation and the degree of skewness by a maximum-likelihood fitting technique. Obesity was defined as a BMI-SDS ≥1.64. Percentile and SDS assessments of weight, height and BMI were made according to the standards of the CDC (14). Waist circumference was measured at the level of the umbilicus with the patient standing and breathing normally. Pubertal maturation was evaluated according to Tanner stages (15). Blood pressure was measured with the auscultatory method using appropriate cuff, in the fasting state and after 20 minutes of rest. Values over 90th percentile according to the age, sex and height tables were considered high (16).

Biochemical Definitions and Insulin Sensitivity Check IndicesPlasma glucose, insulin and lipid levels were measured in blood samples obtained in the morning by venipuncture after an overnight fast. Thyroid, liver and kidney functions were also assessed.  In patients who were found to have a fasting blood glucose (FBG) level of >100 mg/dL, an oral glucose tolerance test (OGTT) was conducted in the morning, after an overnight fast, using a dose of 1.75 g glucose/kg body weight (to a maximum of 75 g) Venous blood samples were obtained at 0, 30, 60, 90 and 120 minute to measure plasma glucose and insulin levels. 

After clotting, the serum was separated and immediately subjected to analysis. The patients were defined as having “impaired glucose tolerance” or “diabetes”, if the glucose level was between 140-200 mg/dL or >200 mg/dL, respectively, in the 2nd hour of the OGTT. 

Hyperinsulinism was defined according to the pubertal stage: prepubertal >15 mU/L; midpuberty (stages 2-4) >30 mU/L (17). Homeostasis model assessment-insulin resistance (HOMA-IR) was calculated according to the formula proposed by Levy et al (18): HOMA-IR=Fasting insulin (mUI/mL) X fasting glucose (mmol/L)/22.5 Plasma concentrations of total cholesterol, high-density lipoprotein-cholesterol (HDL-cholesterol), low-density lipoprotein-cholesterol (LDL-cholesterol), triglycerides and blood glucose were measured using routine enzymatic methods. Fasting HDL-cholesterol and LDL-cholesterol levels were evaluated based on the modified definition criteria of the WHO, Cook and the IDF consensus, and were defined as high, normal or low according to age and sex percentiles (12,13,19). HbA1c was measured by boronic acid affinity chromatography method. 

**Definitions of Metabolic Syndrome**

MS was defined based on the modified WHO criteria adapted for children. The subjects were diagnosed as having MS if they met 3 of the following 4 WHO criteria (19): (1) obesity (BMI >95th percentile for age and sex); (2) abnormal glucose homoeostasis (fasting hyperinsulinemia, impaired fasting glucose, or impaired glucose tolerance); (3) hypertension (systolic blood pressure >95th percentile for age, sex and height); and (4) dyslipidaemia [high triglycerides  (>105 mg/dL in children <10 years of age and >136 mg/dL in children ≥10 years of age), low HDL-cholesterol (<35 mg/dL) or high total cholesterol (>95th percentile)]. 

The risk factors were evaluated according to the modified WHO criteria.The patients were also assessed with the modified Cook criteria (12) and diagnosed as MS if they met at least three of the conditions listed below: (1) triglyceride level >110 mg/dL; (2) HDL-cholesterol level <40 mg/dL;  (3) fasting blood glucose >110 mg/dL; (4) waist circumference >90th percentile for age and sex [normal values for Turkish children (20) were used to assess waist circumference]; and (5) systolic and diastolic blood pressures >90th percentile according to age, sex and height.

The IDF definition of MS for children aged 10 years or older includes BMI>90th percentile for age and sex and presence of two or more of the following findings: (1) triglycerides >150 mg/dL; (2) HDL-cholesterol <40 mg/dL; (3) systolic blood pressure >130 mmHg, diastolic >85 mmHg; and (4) plasma glucose >5.6 mmol/L or >100 mg/dL or known type 2 diabetes (13). 

**Statistical Analysis**

Statistical analyses were performed by SPSS (Statistical Package for Social Sciences) for Windows 15.0 (SPSS Inc., Chicago, IL) program. The data were presented as mean±standard deviation (SD) values. Chi-square test and student’s t-test were used to compare the ratios and the means of the groups, respectively. A p-value of less than 0.05 was considered statistically significant.

## RESULTS

According to the modified WHO criteria, 240 of the 614 patients (39%) were diagnosed with MS. The subjects were between 7 and 18 years of age and after reevaluation with the modified Cook criteria, MS was detected in 209 of them (34%). There were 408 patients aged between 10 and 16 years, of whom 127 (31%) had MS according to the IDF-2007 criteria for children and adolescents. The subjects with a WC>90th percentile constituted 38% of the cases who were below 10 years of age (n=76/198). The overall prevalence of MS in our population according to the IDF criteria was 33%. The clinical and metabolic characteristics of the patients are listed in Table 1. BMI-SDS values in the obese males were significantly higher than those in the females and, males were predominant among MS patients (p<0.001). Although all HbA1c levels were within the normal reference range, males had significantly higher HbA1c levels than females (p=0.006). Mean age, BMI-SDS and weight to height (W/H) ratio of the patients with MS were significantly higher than those of the non-MS patients. In Table 2, MS and non-MS obese patients are compared according to their metabolic characteristics, anthropometric measurements and risk factors for MS. 

The proportion of patients who had high triglyceride and total cholesterol levels were 82% and 18% in the MS group, while these rates were 31% and 15% in the non-MS group. There was a statistically significant difference between the two groups for triglyceride levels (p<0.05), but not for total cholesterol (p>0.05). 

Impaired fasting plasma glucose was detected in 74 out of 240 patients who had MS vs. 17 of 374 non-MS obese patients (p>0.05). Twelve patients had impaired glucose tolerance and 9 of these (75%) had MS.

In 345 patients (56%) who were defined as severely obese, BMI-SDS was above 2.5. The mean age of  the severely obese patients was comparable to that of the subjects who were not severely obese (11.2±2.5 vs. 11.4±2.5 years, p>0.05). MS was detected in 24% (n=65/269) and 51% (n=175/345) of obese and severely obese patients, respectively (p<0.05) (Figure 1A). The numbers of family members who had cardiovascular disease, type 2 diabetes, obesity or hypertension were significantly higher in patients with MS (p<0.05) (Figure 1B). MS was detected in 34% (n=89/259) of children who were exclusively breastfed in the first 6 months of life, while this rate was 52% (n=111/212) in children who were never breastfed (p<0.05) (Figure 1C). There was no significant difference between the birth weights of MS and non-MS patients (Table 2).

When the patients were divided into age groups as 7-11 years (n=344) and 12-18 years (n=270), the prevalence of MS was found to be significantly higher in the second group (114/344 vs. 126/270, p=0.00). Thirty percent (n=90/296) of the prepubertal patients were diagnosed with MS vs. 47% (n=150/318) of the pubertal patients (p<0.05) who were above 7 years old (Figure 1D).

Fasting plasma glucose, triglycerides, HDL-cholesterol, LDL-cholesterol, HbA1c, fasting insulin, and HOMA-IR index of the pubertal and non-pubertal cases are shown in Table 3.

**Figure 1A:**
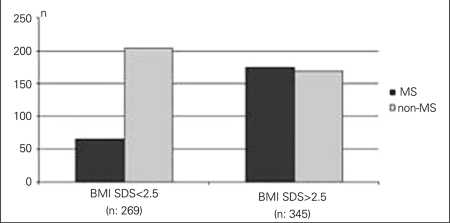
. MS prevalence was 24% and 51% in obese patients who have BMI-SDS<2.5 and BMI-SDS>2.5, respectively (p<0.5)

**Figure 1B:**
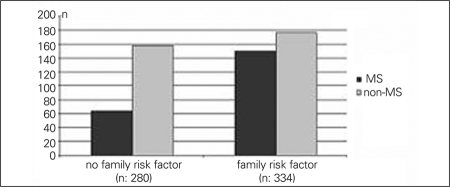
MS prevalence was 22% and 52% in the patients who do not have and  have family risk factors, respectively (p<0.5)

**Figure 1C:**
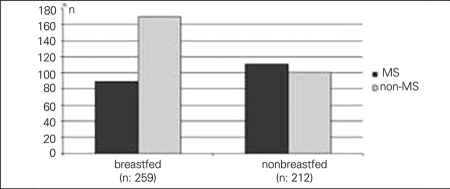
MS prevalence was 34% and 52% in the patients who were exclusively or never breastfed, respectively (p<0.5)

**Figure 1D:**
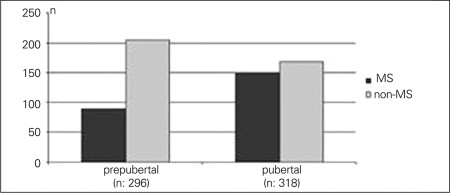
MS prevalance was 30% and 47% in prepubertal and pubertal patients, respectively (p<0.5)

**Table 1:**
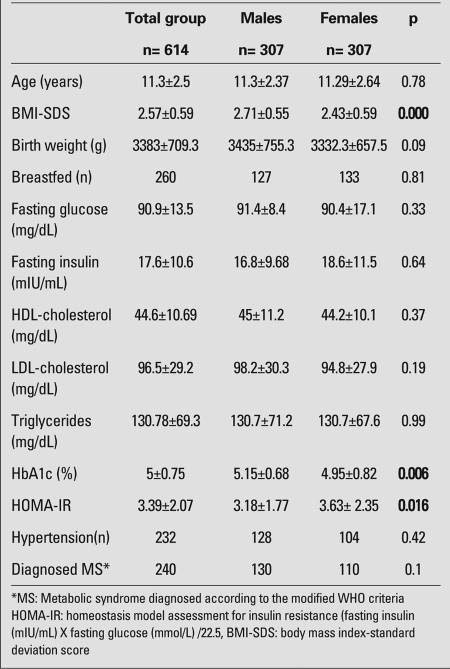
. Clinical characteristics and laboratory parameters of the study population (mean±SD values)

**Table 2:**
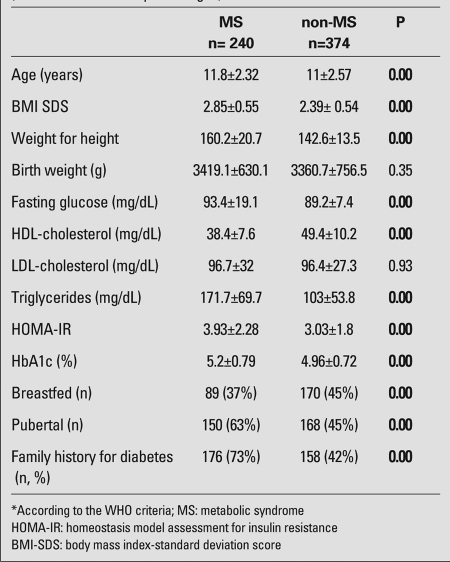
Comparison of risk factors, metabolic characteristics and  anthropometric measurements in MS* and non-MS obese patients (mean±SD values and percentages)

**Table 3:**
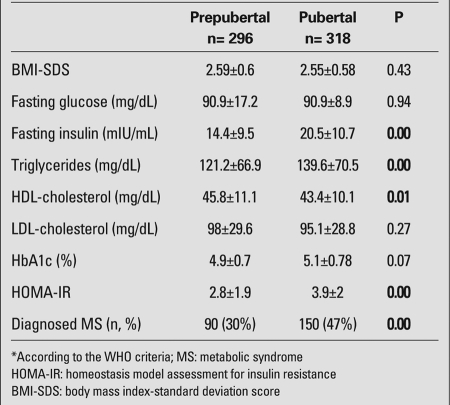
Risk factors and prevalence of MS* by pubertal status (mean±SD)

## DISCUSSION

MS is a cluster of risk factors which is becoming more prevalent concurrent with obesity. Although the prevalence figures differ by diagnostic criteria, the overall prevalence is estimated to be 3-4% (21). According to Cizmecioglu et al (10), 38.8% of obese children in Turkey were diagnosed as having MS based on the WHO criteria, whereas the prevalence declined to 24% when the International Cholesterol Education Panel (NCEP) criteria were used. Miranda et al (22) reported from USA that the MS prevalence was 22% by NCEP criteria, while Isomaa et al (23) found this prevalence to be 42% based on the WHO criteria. In the present study, using the modified WHO criteria, the prevalence of MS was 39% in 614 obese patients between 7 and 18 years of age. When these same patients were reevaluated with the modified Cook criteria, the MS Prevalence decreased to 34%. In addition, we found that 31% of the children aged 10-16 years met the criteria for MS based on the pediatric MS definition of the IDF consensus. The overall prevalence with IDF criteria was 33% and this prevalence was lower than that obtained by other criteria. These different results indicate the necessity to conduct more studies in this field and to revise the diagnostic criteria. 

In relevance to our study, we attempted to present an overview of previous reports on the prevalence of MS in Turkey. Kelestemur et al (7) reported from Kayseri that the prevalence of MS in obese children was 20%. In a study conducted in Ankara city, 4.9% of the 1385 children between 10 and 17 years were obese, and the prevalence of MS in this obese population was estimated to be 21% (8). Atabek et al (9) identified the MS in 27.2% of obese children and adolescents in Konya city.  In our study, the prevalence of MS according to the WHO criteria was similar to that reported by Cizmecioglu et al (11) (39% vs. 38.8%), but higher as compared to the results of the previous studies cited above. This finding can be interpreted as a consequence of the gradually increasing prevalence of MS in children or indicates specific genetic or environmental characteristics of the population in different regions of Turkey. Climate, culture and dietary behaviors are known to vary significantly among the different regions of the country. Meat products and high-fat-containing meals are extensively consumed in some regions, while vegetables and vegetable oil constitute an important food item in other regions. Cold and long winters shorten the duration of outdoor games and enforce the children to a sedentary lifestyle. We consider that these factors affect the MS prevalence as well as the severity and prevalence of obesity in the different regions. In this study, the prevalence of MS was significantly higher in the group which was between 12 and 18 years of age and in the pubertal group. Atabek et al (9) also found that 20% of obese children and 37.6% of adolescents have evidence of MS; however, their patients were not evaluated for pubertal status. There are many reports on the risk factors for MS and, particularly, on insulin resistance, BMI and puberty. It has been shown that when the adjusted insulin resistance according to ethnic origin and the degree of obesity increase, the prevalence of MS also increases (23). A prospective study investigating the cardiovascular risk factors in Finn youth has shown that MS develops in those with high initial insulin levels (24). On the other hand, according to the data of the Bogalusa Heart Study, high BMI, independently from insulin resistance, increases the risk of MS (25). Costa et al (26) observed an association between BMI percentile and a cluster of cardiovascular risk factors, such as increased blood pressure, low HDL-cholesterol, increased triglyceride levels, increased insulin levels, as well as insulin resistance. Pilia et al (27) studied the effect of puberty on insulin resistance and found that the increase in HOMA-IR in obese children at puberty is greater than that in normal-weight children. In this study, we observed significantly higher MS rates in patients in the pubertal group who had high triglyceride, low HDL-cholesterol and high fasting insulin levels as well as high HOMA-IR index and similar BMI-SDS values with prepubertal patients.  Although MS patients have significantly higher BMI-SDS than non-MS patients, puberty is suggested to be one of the major predisposing factors of MS, irrespective of BMI. Osei et al (28) demonstrated that within normal HbA1c limits, there were several anthropometric and metabolic differences among high-risk African-Americans for type 2  diabetes, belonging to the lower and upper HbA1c tertiles. Although all HbA1c levels were in the normal reference range, males had significantly higher HbA1c levels than females in our study. This can be associated with the significantly higher BMI-SDS of the males. Even though the difference is not statistically significant, boys are more prone to be diagnosed with MS, which probably is the result of their higher BMI-SDS and HbA1c levels. However, HOMA-IR values in the females were higher than in males although their BMI and HbA1c levels were lower. It is difficult to interpret these findings and to state that they result from the slightly higher insulin levels of females, a finding which was not statistically significant.Sen et al (29) reported that MS was diagnosed in 38.7% and 49.7% of obese (BMI-SDS: 2-2.5) and severely obese adolescents (BMI-SDS>2.5), respectively. The prevalence of MS was determined as 28.7% in obese adolescents (BMI percentile>95th percentile) in another study, while it was 6.8% in overweight adolescents (BMI: 85-95th percentile) (12). Similarly, MS was detected with a prevalence of 24% and 52% in our obese and severely obese patients. These findings indicate a linear correlation between MS and severity of obesity.  

MS risk is reported to be higher in children of families who have diabetes, heart disease, hypertension and disturbance of lipid metabolism (30,31). We also found that the prevalence of MS is significantly higher in the children who have a family member with diabetes, hypertension, coronary artery disease or hyperlipidemia (Figure 1B). This can be attributed to the effect of genetic factors, but also may indicate similar environmental conditions, dietary habits, socioeconomic status and sedentary lifestyle.

Breast milk is found to be protective against some components of MS (32). It has been reported that the prevalence of obesity increases two-fold in children who were not breastfed as compared to breastfed children, with a concurrent increase in MS prevalence (33). However, Kelishadi et al (34) stated that breast milk has no protective effect against MS, but did not give any data on MS prevalence in non-breastfed children. In our study, the MS prevalence was significantly higher in the non-breastfed group. This result supports the idea that breast milk has a protective effect against obesity and, indirectly, against MS.

Some studies demonstrate that children who have low and high birth weight or a high weight gain in the early postnatal period are at higher risk of developing obesity and MS in older ages, while other studies found that neither gestation week nor birth weight is associated with MS (35,36,37). Our results showed no significant correlation between MS and birth weight and supported the reports stating that birth weight is not being related to MS. However, further studies, particularly comparing children with high or low birth weights, are required to get more conclusive results on the relationships between birth weight and MS.

In conclusion, in parallel to the obesity epidemic, the prevalence of the MS in children and adolescents shows an alarming increase, becoming a growing problem in childhood. Severe obesity, age, pubertal status, presence of family history of cardiovascular disease, type 2 diabetes, obesity or hypertension and not being breastfed during infancy appear to be important risk factors associated with MS in childhood. Further studies are needed to revise the diagnostic criteria in childhood and to reach a consensus on the diagnosis of MS.

**Figure 1B:**
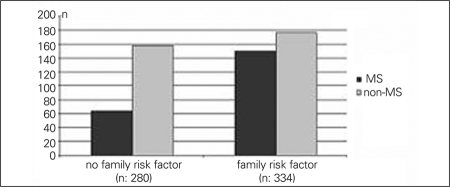
MS prevalence was 22% and 52% in the patients who do not have and  have family risk factors, respectively (p<0.5)
